# A beautiful face is good when we’re judged by others, a moral character is better

**DOI:** 10.1093/scan/nsae071

**Published:** 2024-10-17

**Authors:** Julia Baum, Rasha Abdel Rahman

**Affiliations:** Faculty of Life Sciences, Department of Psychology, Humboldt-Universität zu Berlin, Berlin 10099, Germany; Humboldt-Universität zu Berlin, Faculty of Philosophy, Berlin School of Mind and Brain, Berlin 10099, Germany; Faculty of Life Sciences, Department of Psychology, Humboldt-Universität zu Berlin, Berlin 10099, Germany; Humboldt-Universität zu Berlin, Faculty of Philosophy, Berlin School of Mind and Brain, Berlin 10099, Germany

**Keywords:** face processing, attractiveness, halo of beauty, social-emotional person knowledge, social judgments, event-related potentials

## Abstract

Moral beauty, reflected in one’s actions, and facial beauty both affect how we are judged. Here, we investigated how moral and facial beauty interact to affect social judgments and emotional responses, employing event-related brain potentials (ERPs). All participants (female) associated positive, neutral, or negative verbal information with faces scoring high or low on attractiveness and performed ratings of the faces as manipulation checks. In a separate test phase, the faces were presented again, and participants made valenced social judgments of the persons. Results show a dominance of moral beauty in valenced social judgments as well as ERPs related to reflexive and evaluative emotional responses (early posterior negativity and late positive potential), whereas facial attractiveness mattered little. In contrast, facial attractiveness affected visual processing (N170). Similarly, relatively shallow impressions of attractiveness and likability that require no knowledge about the person were influenced by both facial attractiveness and social-emotional information. This pattern of dominant effects of social-emotional information regardless of attractiveness shows that when it comes to our emotional responses and social judgments, moral beauty is what matters most, even in the face of physical beauty.

Facial beauty and moral beauty both matter for our social impressions. Previous research has focused on either source of information in isolation, but little is known about how these different sources of visual and nonvisual information interact. This study investigated the combined effects of moral beauty and facial beauty on social judgments and emotional responses. Using event-related brain potentials (ERPs) to identify the underlying neurocognitive processes, we tested temporally resolved effects on face processing from visual processing to reflexive emotional brain responses mid-latency, and later, more evaluative responses.

Moral beauty relates to a person’s character and social behavior and can be derived from verbally transmitted, social-emotional information. It changes the affective value of people via mechanisms of evaluative learning (Goodwin 2015, Xu et al. 2016, Ferrari et al. 2020). Social-emotional information has been shown to influence the perception and evaluation of others (Abdel Rahman 2011, Tsukiura and Cabeza 2011b, Suess et al. 2013, Cui et al. 2019, Maier et al. 2022), ranging from affecting the chances of when a face is consciously perceived (Anderson et al. 2011, Mo et al. 2016, Rabovsky et al. 2016, Eiserbeck and Abdel Rahman 2020, Eiserbeck et al. 2024), over indirect evaluations of facial features (Hassin and Trope 2000), trustworthiness (Mattarozzi et al. 2019, Eiserbeck and Abdel Rahman 2020), attractiveness (Nisbett and Wilson 1977, He et al. 2024), and likability, to explicit social judgments of the person and their social character (Bliss-Moreau et al. 2008, Todorov and Olson 2008, Suess et al. 2013, Baum and Abdel Rahman 2020, 2021, Baum et al. 2020). Social-emotional information is spontaneously processed in face perception as reflected in neural regions associated with social cognition and emotion, including emotional responses in the anterior insula known to process disgust-related emotional pictures (Todorov et al. 2007).

Facial beauty, on the other hand, is directly visible in a person’s physical appearance. We attribute more positive traits like trustworthiness, friendliness, and social competence to attractive individuals (the halo effect and beautiful-is-good effect), whereas individuals scoring low in attractiveness are seen in a less positive light and face even negative prejudices (Dion et al. 1972, Eagly et al. 1991, Langlois et al. 2000, Zebrowitz and Montepare 2008, Oh et al. 2023). Despite its low diagnostic value we feel confident in the validity of our evaluations based on physical appearance (Hassin and Trope 2000, Olivola et al. 2014). Neurocognitive evidence suggests that the neural mechanisms for judging facial beauty and moral beauty (e.g. from depictions of scenes containing moral actions) are highly similar, suggesting that biases from attractiveness are emotionally and socially relevant (Tsukiura and Cabeza 2011b, Wang et al. 2014, Mo et al. 2016, Cui et al. 2019). Impressions of high versus low attractiveness have been found in differential effects related to approach and avoidance in the medial orbitofrontal cortex and insular cortex, respectively (Tsukiura and Cabeza 2011a, 2011b).

We investigate the interplay of moral and facial beauty in well-described ERP components from earlier to later face processing. Visual perception is reflected in the P1 and N170 components, with the P1 reflecting low-level processing like perceived contrast [100 ms after face presentation; Haynes et al. (2003)], and the N170 reflecting higher-level configural encoding [170 ms, occipito-temporal regions; Eimer (2011)]. Both have been found to be sensitive to facial expression analysis and emotion processing, although with less stable emotion effects compared to later ERPs (Meeren et al. 2005, Thierry et al. 2007, Dering et al. 2011, Hinojosa et al. 2015, Schindler and Bublatzky 2020, Schindler et al. 2023). Mid-latency, we test effects on fast and reflexive emotional processing in the early posterior negativity [early posterior negativity (EPN), 200–350 ms, occipito-temporal regions]. Late in the processing, we test effects on slower, higher-level evaluation of emotional meaning in the late positive potential [late positive potential (LPP), 400–600 ms, centro-parietal regions]. Original findings related to emotional picture processing, i.e. pleasant or unpleasant visual scenes or objects, as well as friendly or threatening faces in comparison to relatively neutral stimuli found that emotionally arousing content elicited modulations starting from about 100 to 150 ms onwards with modulations of the EPN and LPP. Findings suggest that affective meaning is processed independent of physical picture features, and even when stimuli are presented very briefly (Junghöfer et al. 2001, Schupp et al. 2004). Both EPN and LPP were found to be primarily driven by the arousal dimension of emotional content such that effects of affective arousal can be similarly found for positive and negative valenced stimuli (e.g. Dolcos and Cabeza 2002, Schupp et al. 2003, 2006). While EPN effects seem to occur relatively reflexively, LPP modulations are more sensitive to the meaning or relevance of emotional stimuli, depending on the current task and motivation (Schupp et al. 2000, Schacht and Sommer 2009, Rellecke et al. 2011, 2012, Blechert et al. 2012).

Knowing about the moral beauty of a person affects ERPs during face processing. Reported effects by associated social-emotional information start in ERP components related to visual processing (N170), which may reflect attention effects or altered visual perception (Luo et al. 2016, Giménez-Fernández et al. 2020, Baum and Abdel Rahman 2021, Schindler et al. 2021). EPN effects show that social-emotional information affects reflexive emotional processing (Abdel Rahman 2011, Kissler and Strehlow 2017, Baum and Abdel Rahman 2020, 2021), possibly without attention to the information (Suess et al. 2013, Xu et al. 2016, Eiserbeck et al. 2024), yet at this stage effects may depend on the saliency of the stimulus and the available processing capacities (Schindler et al. 2021). LPP modulations show effects on more controlled, elaborate person evaluation (Abdel Rahman 2011, Xu et al. 2016, Kissler and Strehlow 2017, Baum and Abdel Rahman 2020, 2021, Baum et al. 2020). EPN and LPP effects seem primarily driven by arousal. Although few studies even included positive social-emotional information, earlier ERP effects have more often been found for negative information, whereas effects in the LPP have been found for positive and negative stimuli.

Facial beauty has likewise been shown to affect ERPs during face processing. Modulations by different levels of perceived attractiveness start with the P1, P2, and N170 but have been reported in different directions (Schacht and Sommer 2009, Marzi and Viggiano 2010, Zhang and Deng 2012, Trujillo et al. 2014, Hahn et al. 2016). Since these components are sensitive to low-level differences in visual stimuli, it is possible that the inconsistent findings are due to specific perceptual stimulus features. EPN modulations have been found with enhanced amplitudes for attractive compared to less attractive faces (Werheid et al. 2007, Marzi and Viggiano 2010, Wiese et al. 2014, Thiruchselvam et al. 2016). The LPP was shown to be modulated by attractiveness, albeit in different directions. While some findings show that both high and low attractive faces elicit LPP effects in comparison to more neutral or intermediate faces, others show enhanced amplitudes for attractive compared to less attractive faces (Werheid et al. 2007, Schacht et al. 2008, Marzi and Viggiano 2010, Revers et al. 2023).

Here, we examined how behavioral and ERP effects of social-emotional information are influenced by facial attractiveness in a social judgment task (Fig. 1). If effects are modulated, we expect modulations by the high or low end of attractiveness, possibly related to (in)congruency with information. We included intermediate attractive faces only as fillers and always paired with neutral information (see supplementary material, SI-page 14 f for additional analyses including fillers). This furthermore balanced the instances of extremes of attractiveness and information in the context of the experiment. Additionally, participants rated their impressions of attractiveness and spontaneous likeability as manipulation checks both before and after the person-related information was given. These ratings allow us to investigate effects when social-emotional information is not or only indirectly relevant.

**Figure 1.**
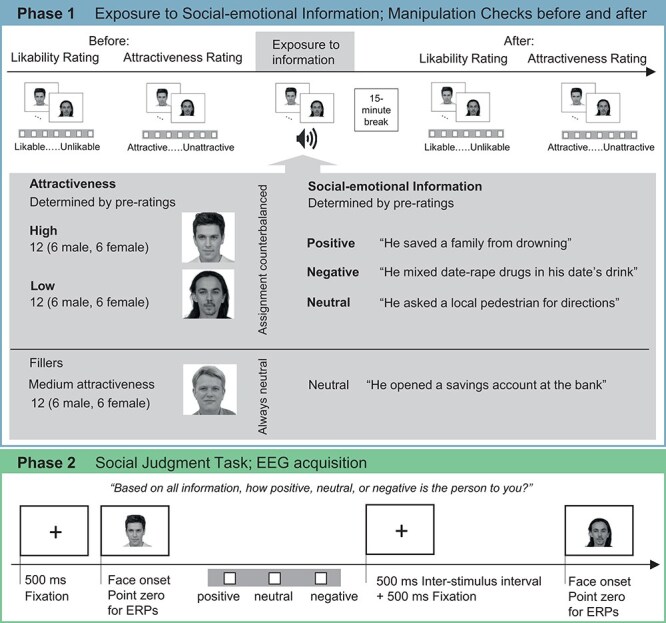
Schematic overview of the study phases and experimental manipulations: In Phase 1 likability and attractiveness of the faces was rated before and after praticipants were exposed to social-emotional information about the faces; in Phase 2 the faces were presented without the associated social-emotional information and the EEG of the participants was acquired while they judged the persons as positive, neutral, or negative, taking all available information into account (AI generated example faces are shown; Academic Dataset by Generated Photos https://generated.photos/datasets).

We expected effects of positive and negative information on valenced social judgments and modulations of the EPN and LPP, reflecting key processes involved in social-emotional face and person evaluation, and where previous research has found effects of moral and facial beauty but has not studied their interaction. Associated emotional information may already influence the N170 (see Schindler et al. 2021), yet visual processing (P1, N170) may be most influenced by attractiveness; however, this might be partly due to low-level differences in the facial stimuli. Crucially, our investigation focuses on whether, how, and when facial attractiveness modulates behavioral and neurocognitive effects of associated social-emotional information.

## Method

### Participants

The sample size was planned according to the requirements of the counterbalancing and based on power analyses, see SI-page 1 in supplementary material. The dataset consists of 24 female participants (mean age = 23.46 (SD = 4.78), 23 right-handed). We invited females only to avoid differences in the perception of attractiveness in male and female faces (see e.g. Tsukiura and Cabeza 2011a, 2011b). Five participants had to be replaced with new participants (reasons: one mistake in the counterbalancing, one could not remember the gist of the person information, two did not press buttons, and one had no vision on one eye). Participants were (de) briefed about the procedures and signed informed consent. The study was approved by the local ethics committee.

## Materials

Thirty-six unfamiliar faces with neutral facial expressions were selected from a database previously used to study effects of attractiveness (Schacht et al. 2008). Based on independent pre-ratings (see SI-page 1 in supplementary material), we selected 12 high and 12 low attractiveness target faces, and 12 filler faces of medium attractiveness, with half male and half female faces, respectively. Photographs were grayscale, adjusted in luminance for similar brightness and placed on a light blue background (2.7 × 3.5 cm on a 19”, 60 Hz, 1280 × 1024 monitor, viewing distance 70 cm; equating to a visual image size of app. 2.5°) to assimilate low-level features.

Faces were associated with social-emotional information that was either positive, negative, or neutral in valence (for pre-ratings see SI-page 2 in supplementary material). The information referred to the person’s social and moral character, e.g. positive: “He saved a family of refugees from drowning”, negative: “He mixed date-rape drugs in his date’s drink” (or was neutral: “He showed the new collection to the customers”). All 36 sentences were recorded by the same male speaker.

Taken together, each attractiveness (high, low) by information (positive, negative, neutral) condition consisted of four target face stimuli. By counterbalancing the assignment of information to target faces between participants, we ensured that across participants each face was associated equally often with each of the three information levels. Please note that the assigned information to each face was consistent within a participant. The twelve filler faces were always paired with neutral information.

### Procedure

The within-subject design consisted of two phases, a well-established procedure in studying face perception and social-emotional information (Abdel Rahman 2011, Baum and Abdel Rahman 2020, Eiserbeck and Abdel Rahman 2020, Suess et al. 2013). In Phase 1, the experiment started with a likability and an attractiveness rating of all 36 presented faces as manipulation checks, and the order of the ratings was counterbalanced between participants [7-point scales adopted from the Self-Assessment Manikin; (Bradley and Lang 1994), from very likable to very unlikable, and very high to very low]. For the exposure to social-emotional information, participants saw each face and were presented with auditory information about them. Presented were blocks of nine faces that included all experimental conditions and three fillers. In each trial, the face was presented for 6 s and each face was repeated five times in total (but not directly repeating). To ensure participants paid attention, they occasionally answered simple yes-or-no questions referring to the information interspersed between blocks (e.g. Is the behavior of this person common?). After a 15-min break, Phase 1 concluded with the same likability and attractiveness ratings of all faces, serving as before vs. after information exposure manipulation checks. In total, Phase 1 had 324 trials per participant.

In Phase 2, the faces were shown in isolation and participants performed the social judgment task as the main task and their EEG was recorded. They judged the persons referring to their social characteristics based on all available information (“Based on all information, how positive, neutral, or negative is this person to you?”, 3-point scale). We chose this explicit judgment to compare the results with other studies using a similar social judgment task, but did not manipulate the facial appearance (e.g. Baum et al. 2018; Baum and Abdel Rahman 2020, 2021, Schindler et al. 2021). Each trial started with a 500-ms fixation cross, and faces were presented until button press or for 2 s, followed by a 500-ms interstimulus interval. The social judgment task was repeated 20 times in blocks of all 36 faces separated by self-paced breaks (resulting in 80 trials per participant per condition). Participants were told that repetitions are necessary for EEG measurements. In total, Phase 2 had 792 trials per participant.

The direction of answer scales was counterbalanced between participants, i.e. half of the participants had scales and buttons range from positive (left) to negative (right), and the other half vice versa. After the experiment, participants were asked to write down the gist of the information associated with each face to check for sufficient acquisition of the information.

### EEG data recording and preprocessing

The EEG was recorded from 62 Ag/AgCl scalp electrodes as specified by the extended 10–20 system, referenced to the left mastoid with FCz as the ground electrode (see SI-page 3 for setup in supplementary material). The impedance was kept below 5kΩ. EEG data were recorded at a sampling rate of 5 kHz and down-sampled to 500 Hz using a low cutoff of 0.016 Hz and a high cutoff of 1000 Hz. Horizontal and vertical electrooculograms were obtained with peripheral electrodes at the left and right canthi of both eyes, and above and below the left eye. A short calibration procedure traced individual eye movements after the experiment, which were later used to correct for eye movement artefacts.

Offline, the continuous EEG was transformed to average reference (see SI-page 3 for all electrodes in supplementary material), and high-pass filtered at 0.1 Hz and low-pass filtered at 30 Hz pass-band edge [zero-phase FIR-filter with transition band width of 0.1 Hz and cutoff frequency (−6 dB): 0.05, 30.05 Hz, EEGlab-toolbox version 13_5_4b; Delorme and Makeig (2004)]. Using BESA (Berg and Scherg 1991), we removed artefacts due to eye movements by applying a spatiotemporal dipole modeling procedure for each participant individually. Trials with remaining artefacts were rejected, i.e. trials with amplitudes over ±200 µV, changing more than 50 µV between samples or more than 200 µV within single epochs, or containing baseline drifts. Error- and artefact-free EEG data were segmented into epochs of 1 s, starting 200 ms prior to stimulus onset, with a 200-ms pre-stimulus baseline. For EEG analysis, an average of 78 trials per participant remained (range: 70-80) in each condition. Overall, 97% of trials were kept (high attractive: positive 1869, negative 1877, neutral 1844, low attractive: positive 1870, negative 1876, neutral 1860). Trials where no judgment was given or latencies below 150 ms were excluded (in the social judgment task that were 323 excluding fillers, leaving a total of 11 196 trials).

### Data analysis

ERP analyses focused on the EPN component (electrode sites PO7, PO8, PO9, PO10, TP9, TP10, 200–350 ms after face stimulus onset), and the LPP component (electrode sites Pz, CPz, POz, P3, P4, 400–600 ms), according to previous findings of effects of social-emotional information (Abdel Rahman 2011; Baum et al. 2018, Baum and Abdel Rahman 2020, 2021), emotion effects (Schupp et al. 2003), and attractiveness effects (Werheid et al. 2007, Schacht et al. 2008, Marzi and Viggiano 2010, Wiese et al. 2014, Thiruchselvam et al. 2016). Additionally, we analyzed effects during early visual face processing in the P1 (PO3, PO4, O1, O2, 80–120 ms) and the N170 (P7, P8, PO7, PO8, 130–200 ms), also based on previous research (Abdel Rahman and Sommer 2012, Hinojosa et al. 2015, Baum and Abdel Rahman 2020, 2021, Schindler et al. 2021). Amplitudes were averaged over the respective regions of interest and time windows on single-trial level.

We performed mixed-effects regression models on single-trial data of ERPs and behavioral measures in R (Bates et al. 2015, Frömer et al. 2018). We calculated the effects of positive information (positive vs. neutral) and negative information (negative vs. neutral) each in interaction with attractiveness in separate 2 × 2 models. We analyzed the effects of positive and negative information separately because they have resulted in different outcomes in our previous studies (e.g. Baum and Abdel Rahman 2021). We specified each model with the fixed effects for the experimental factors “information” (positive or negative vs. neutral; with neutral as the reference level) and “attractiveness” (high vs. low; with low as the reference level) and their interaction. For the manipulation checks (likability rating and attractiveness rating), we nested the factors information and attractiveness with the factor levels of “phase” (before, after). All factors were modeled as repeated contrasts that compare the means of factor levels to the respective reference level (Schad et al. 2020). For continuous dependent variables (ERPs, RTs), we used linear mixed models (LMMs; *lme4* v.1.1-26; RTs were reciprocally transformed to −1000/RT; amplitudes were normally distributed), and for the social judgments and ratings, we used cumulative link mixed models (CLMMs; *ordinal* v.2022.11-16). We fitted models with by-subject random intercept and slopes, and by-face random intercepts. If necessary, random-slopes correlation parameters were set to zero and slopes explaining zero variance were omitted to achieve convergence and avoid overparameterization (Bates et al. 2015). We test the significance of fixed effects coefficients (*P*-value < .05) via the *summary* command (with Satterthwaite statistics for LMMs and Wald statistics for CLMMs). We report unstandardized fixed effects coefficients as effect sizes (*b* with standard error SE). Separate comparisons were performed using *emmeans* (v.1.4.6, Lenth 2020) with false discovery rate-adjusted *P*-values (Benjamini and Hochberg 1995). Data and code are available online (https://osf.io/e529z/).

## Results

### Social judgment task (Phase 2): social judgments and brain responses as a function of social-emotional information and attractiveness

#### Behavioral results

**Figure 2.**
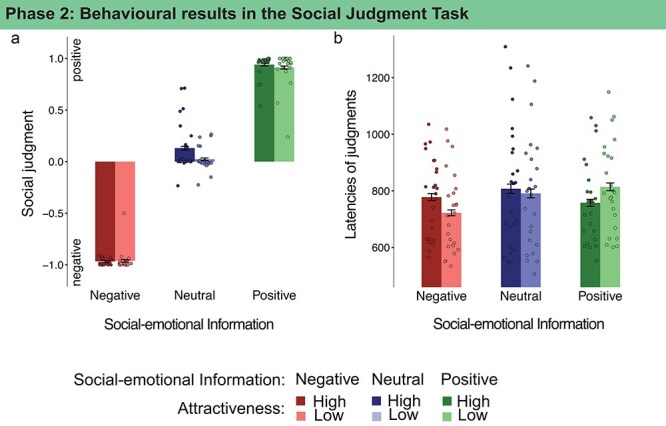
Social Judgment Task (Phase 2): (a) Social judgments were dominated by the associated social-emotional information, and attractiveness did not modulate judgments; (b) latencies of social judgments were affected by the congruency of social-emotional information and attractiveness, with faster judgments of high attractive faces associated with positive information and faster judgments of low attractive faces associated with negative information (dots represent individual participant means, error bars show 95% confidence intervals).

Faces were presented and participants judged the persons based on all available information on a valence scale (Fig. 2a). Faces associated with negative information were judged as more negative persons compared to neutral information, regardless of attractiveness (Table 1). Faces associated with positive information were judged as more positive persons compared to faces associated with neutral information, also regardless of attractiveness (Table 1).

**Table 1.** Mixed model summary statistics and separate comparisons show effects of positive information and negative information each in interaction with attractiveness on social judgments

Social judgments				
*Coefficient*	*b*	SE	*z*	*P*
High vs. low attractiveness	0.41	0.36	1.11	.27
Negative vs. neutral information	−8.15	0.44	−18.61	**<.001**
Positive vs. neutral information	6.76	0.39	17.46	**<.001**
Negative vs. neutral info × high vs. low att	−0.29	0.49	−0.59	.56
Positive vs. neutral info × high vs. low att	−0.68	0.70	−0.98	.33
*Separate comparisons*	*b*	SE	*z*	*P*
High attractiveness: negative vs. neutral info	−8.29	0.53	−15.43	**<.001**
High attractiveness: positive vs. neutral info	6.42	0.45	14.16	**<.001**
High attractiveness: negative vs. positive info	−14.71	0.53	−27.58	**<.001**
Low attractiveness: negative vs. neutral info	−8.00	0.46	−17.24	**<.001**
Low attractiveness: positive vs. neutral info	7.10	0.58	12.25	**<.001**
Low attractiveness: negative vs. positive info	−15.11	0.83	−18.13	**<.001**
Negative information: high vs. low Att	0.44	0.51	0.87	.43
Positive information: high vs. low Att	0.05	0.66	0.07	.94
Neutral information: high vs. low att	0.73	0.41	1.77	.098
*Formula of converging model*	Judgments ∼ information × attractiveness + (information × attractiveness| subject) + (1 | face)			

*Note. *× stands for interaction. Double bars in random effects terms set correlation parameters to zero. Separate comparisons’ *P*-values were FDR corrected for nine tests. *P*-values < .05 are printed in bold.

For the latencies of social judgments, the effects of information and attractiveness interacted such that congruent conditions lead to faster judgments (Fig. 2b). Faces associated with negative information were judged faster when they were low in attractiveness, and faces associated with positive information were judged faster when they were high in attractiveness (Table 2).

**Table 2.** Linear mixed model summary statistics and separate comparisons show effects of positive information and negative information each in interaction with attractiveness on latencies of social judgments

Latencies of social judgments [-1000 / latency (ms)]				
*Coefficient*	*b*	SE	*t*	*P*
Intercept (grand mean)	−1.44	0.05	−28.50	**<.001**
High vs. low attractiveness	0.011	0.02	0.49	.63
Negative vs. neutral information	0.008	0.03	0.27	.79
Positive vs. neutral information	0.056	0.03	1.74	.095
Negative vs. neutral info × high vs. low att	0.056	0.02	2.28	**.032**
Positive vs. neutral info × high vs. low att	−0.11	0.02	−5.57	**<.001**
*Separate comparisons*	*b*	SE	*t*	*P*
High attractiveness: negative vs. neutral info	0.035	0.03	1.15	.39
High attractiveness: positive vs. neutral info	−0.00	0.03	−0.00	.99
High attractiveness: negative vs. positive info	0.034	0.04	0.80	.548
Low attractiveness: negative vs. neutral info	−0.020	0.03	−0.66	.58
Low attractiveness: positive vs. neutral info	0.11	0.03	3.32	**.010**
Low attractiveness: negative vs. positive info	−0.13	0.04	−2.98	**.010**
Negative information: high vs. low att	0.085	0.03	3.08	**.010**
Positive information: high vs. low att	−0.082	0.03	−3.08	**.010**
Neutral information: high vs. low att	0.030	0.03	1.17	.39
*Formula of converging model*	Latencies ∼ information × attractiveness + (information × attractiveness|| subject) + (1 | face)			

*Note. *× stands for interaction. Double bars in random effects terms set correlation parameters to zero. Separate comparisons’ *P*-values were FDR corrected for nine tests. *P*-values < .05 are printed in bold.

#### Event-related brain potentials

**Figure 3.**
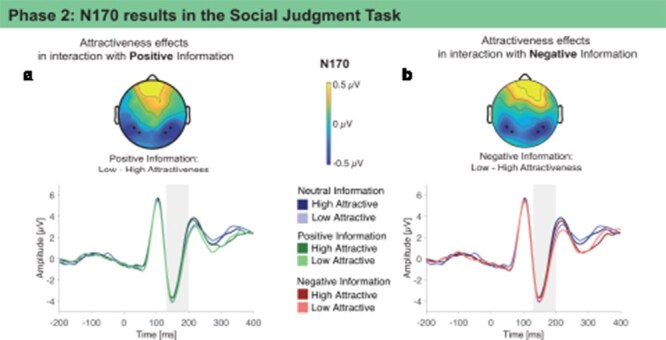
N170 (130–200 ms) modulations show attractiveness effects for positive and negative social-emotional information during the Social Judgment Task in Phase 2 (grand average ERPs are shown at the respective region of interest shown as black dots in the topographies, scalp topographies show the effects as differences between conditions in the time windows shaded in gray).

**P1 and N170**. For effects on visual face perception, we investigated the P1 and the N170. No modulations were found in the P1 (see SI-page 3 in supplementary material). The N170 was enhanced for low attractive faces compared to high attractive faces (Table 3, Fig. 3). Trends for interaction effects of negative and positive information with attractiveness showed, if anything, enhanced N170 amplitudes for low vs. high attractive faces in the negative and positive information condition, but not in the neutral condition.

**Table 3.** Linear mixed model summary statistics and separate comparisons show effects of positive information and negative information each in interaction with attractiveness on the N170

N170				
*Coefficient*	*b*	SE	*t*	*P*
Intercept (grand mean)	−1.49	0.61	−2.45	**.021**
High vs. low attractiveness	0.49	0.17	2.82	**.009**
Negative vs. neutral information	−0.11	0.11	−1.00	.33
Positive vs. neutral information	−0.08	0.13	−0.60	.55
Negative vs. neutral info × high vs. low att	0.37	0.22	1.69	.098
Positive vs. neutral info × high vs. low att	0.43	0.22	1.95	.051
*Separate comparisons*	*b*	*SE*	*t*	*p*
High attractiveness: negative vs. neutral info	0.08	0.16	0.52	.78
High attractiveness: positive vs. neutral info	0.14	0.17	0.81	.63
High attractiveness: negative vs. positive info	−0.06	0.17	−0.34	.83
Low attractiveness: negative vs. Neutral info	−0.29	0.16	−1.88	.18
Low attractiveness: positive vs. neutral info	−0.29	0.17	−1.73	.20
Low attractiveness: negative vs. positive info	−0.00	0.17	−0.01	.99
Negative information: high vs. low att	0.60	0.22	2.77	**.036**
Positive information: high vs. low att	0.65	0.22	3.03	**.033**
Neutral information: high vs. low att	0.22	0.22	1.04	.55
*Formula of converging model*	N170 ∼ information × attractiveness + (pos-neu + high-low + neg-neu-high-low|| subject) + (1 | face)			

*Note. *× stands for interaction. Double bars in random effects terms set correlation parameters to zero. Separate comparisons’ *P*-values were FDR corrected for nine tests. *P*-values < .05 are printed in bold.

**Figure 4.**
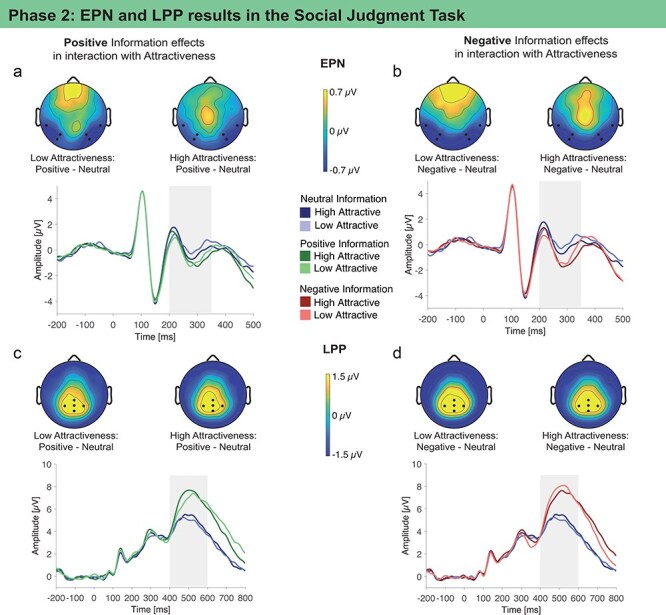
EPN (200–350 ms) and LPP (400–600 ms) modulations show dominant effects of positive and negative social-emotional information that were largely unaffected by attractiveness during the Social Judgment Task in Phase 2 (grand average ERPs are shown at the respective region of interest shown as black dots in the topographies, scalp topographies show the effects as differences between conditions in the time windows shaded in gray).

**EPN**. For fast and reflexive emotional processing we investigated the EPN component (Fig. 4a and b). The EPN was enhanced for faces associated with negative information compared to neutral and for faces associated with positive information compared to neutral (Table 4). Attractiveness did not modulate EPN effects (Table 4).

**Table 4.** Linear mixed model summary statistics and separate comparisons show effects of positive information and negative information each in interaction with attractiveness on the EPN

EPN				
*Coefficient*	*b*	SE	*t*	*P*
Intercept (grand mean)	−0.11	0.55	−0.20	.84
High vs. low attractiveness	−0.06	0.36	−0.17	.87
Negative vs. neutral information	−0.88	0.12	−7.45	**<.001**
Positive vs. neutral Information	−0.64	0.15	−4.30	**<.001**
Negative vs. neutral info × high vs. low att	0.05	0.29	0.19	.85
Positive vs. neutral info × high vs. low att	0.21	0.22	0.99	.33
*Separate comparisons*	*b*	SE	*t*	*P*
High attractiveness: negative vs. neutral info	−0.85	0.19	−4.53	**<.001**
High attractiveness: positive vs. neutral info	−0.53	0.18	−2.88	**.012**
High attractiveness: negative vs. positive info	−0.32	0.21	−1.48	.26
Low attractiveness: negative vs. neutral info	−0.90	0.19	−4.83	**<.001**
Low attractiveness: positive vs. neutral Info	−0.74	0.18	−4.05	**<.001**
Low attractiveness: negative vs. positive info	−0.16	0.22	−0.75	.68
Negative information: high vs. low att	−0.10	0.41	−0.24	.88
Positive information: high vs. low att	0.06	0.39	0.16	.88
Neutral information: high vs. low att	−0.15	0.39	−0.39	.88
*Formula of converging model*	EPN ∼ information × attractiveness + (information × attractiveness|| subject) + (1 | face)			

*Note. *× stands for interaction. Double bars in random effects terms set correlation parameters to zero. Separate comparisons’ *P*-values were FDR corrected for nine tests. *P*-values < .05 are printed in bold.

**LPP**. For slower and more elaborate evaluative processing, we investigated the LPP component (Fig. 4c and d). The LPP was enhanced for faces associated with negative compared to neutral information and for faces associated with positive compared to neutral information (Table 5). Attractiveness did not modulate LPP effects of positive information. Attractiveness interacted with the effect of negative information such that LPP amplitudes for negative associated information were most pronounced for low attractive faces, suggesting an effect of congruency (Table 5).

**Table 5.** Linear mixed model summary statistics and separate comparisons show effects of positive information and negative information each in interaction with attractiveness on the LPP

LPP				
*Coefficient*	*b*	SE	*t*	*P*
Intercept (grand mean)	5.96	0.58	10.26	**<.001**
High vs. low attractiveness	0.06	0.18	0.32	.75
Negative vs. neutral information	2.04	0.21	9.91	**<.001**
Positive vs. neutral information	1.74	0.16	10.74	**<.001**
Negative vs. neutral info × high vs. low att	−0.60	0.26	−2.31	**.027**
Positive vs. neutral info × high vs. low att	0.16	0.23	0.69	.49
*Separate comparisons*	*b*	SE	*t*	*P*
High attractiveness: negative vs. Neutral info	1.74	0.24	7.15	**<.001**
High attractiveness: positive vs. Neutral info	1.82	0.20	9.10	**<.001**
High attractiveness: negative vs. Positive info	−0.08	0.27	−0.29	.77
Low attractiveness: negative vs. Neutral info	2.34	0.24	9.62	**<.001**
Low attractiveness: positive vs. neutral info	1.66	0.20	8.30	**<.001**
Low attractiveness: negative vs. positive info	0.68	0.27	2.52	**.030**
Negative information: high vs. low att	−0.39	0.24	−1.64	.16
Positive information: high vs. low att	0.37	0.23	1.58	.15
Neutral information: high vs. low att	0.20	0.23	0.88	.43
*Formula of converging model*	LPP ∼ information × attractiveness + (information × attractiveness|| subject) + (1 | face)			

*Note. *× stands for interaction. Double bars in random effects terms set correlation parameters to zero. Separate comparisons’ *P*-values were FDR corrected for nine tests. *P*-values < .05 are printed in bold.

### Manipulation checks (Phase 1)

#### Likability ratings before and after information exposure

**Figure 5.**
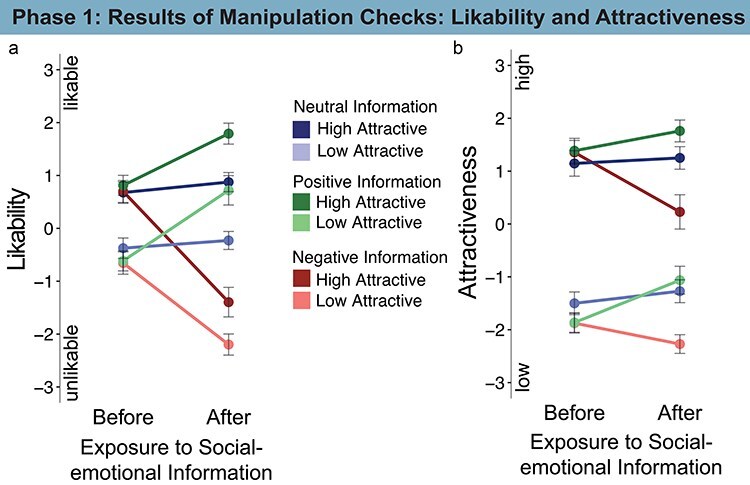
Likability (a) and attractiveness (b) ratings of the faces in Phase 1 were determinded by attractiveness before social-emotional information was acquired, whereas, after exposure to social-emotional information ratings were influenced by both attractiveness and social-emotional information.

Before information exposure, we replicated effects from the literature showing that faces high in attractiveness were more likable than faces low in attractiveness (Fig. 5, Table 6). The interaction of attractiveness with information before information was given was unexpected, because the assignment of faces to information conditions was counterbalanced and thereby controlled (see SI-page 4 f for additional analyses in supplementary material showing that this pertains the neutral condition).

**Table 6.** Mixed model summary statistics show effects of positive knowledge and negative knowledge each in interaction with attractiveness on likability ratings before and after knowledge acquisition

Likability rating				
*Coefficient*	*b*	SE	*z*	*P*
Phase (before vs. after)	0.30	0.19	1.61	.11
Before information exposure				
High vs. low attractiveness	2.92	0.43	6.86	**<.001**
Negative vs. neutral information	−0.25	0.21	−1.18	.24
Positive vs. neutral information	−0.07	0.23	−0.28	.78
Negative vs. neutral info × high vs. low att	0.80	0.44	1.80	.07
Positive vs. neutral info × high vs. low att	0.94	0.46	2.07	**.039**
After information exposure				
High vs. low attractiveness	2.29	0.48	4.79	**<.001**
Negative vs. neutral information	−4.97	0.45	−10.97	**<.001**
Positive vs. neutral information	2.62	0.59	4.42	**<.001**
Negative vs. neutral info × high vs. low att	−0.65	0.57	−1.15	.25
Positive vs. neutral info × high vs. low att	0.09	0.56	0.17	.87
*Formula of converging model*	Rating ∼ phase/ information × attractiveness + (1 + phase/ information × attractiveness| subject) + (1 | face)			

*Note. *× stands for interaction. The / in the model formula denotes nesting. *P*-values < .05 are printed in bold.

After information exposure, likability ratings showed main effects of information and attractiveness, and no interactions (Fig. 5, Table 6). Faces associated with positive information and faces high in attractiveness were rated more likable than those with neutral information and low attractiveness, respectively. Faces associated with negative information and faces low in attractiveness were rated less likable than those with neutral information and high attractiveness, respectively.

#### Attractiveness ratings before and after information exposure

We manipulated the attractiveness of faces based on pre-ratings as high or low and validated our manipulation with the current sample.

Before information exposure, results confirmed that faces were clearly perceived as differentially attractive (Fig. 5, Table 7). Unexpectedly, we found interaction effects of attractiveness and information before participants were exposed to information. As was the case for the likability rating, these effects were driven by the neutral information condition (see SI-page 4 f in supplementary material).

**Table 7.** Mixed model summary statistics and follow-up tests show effects of positive information and negative information each in interaction with attractiveness on attractiveness ratings before and after information exposure

Attractiveness rating				
*Coefficient*	*b*	SE	*z*	*P*
Phase (before vs. after)	0.03	0.12	0.26	.80
Before information exposure				
High vs. low attractiveness	6.18	0.57	10.76	**<.001**
Negative vs. neutral information	−0.25	0.20	−1.29	.20
Positive vs. neutral information	−0.12	0.21	−0.55	.58
Negative vs. neutral info × high vs. low att	1.28	0.41	3.14	**.002**
Positive vs. neutral info × high vs. low att	1.40	0.43	3.25	**.001**
After information exposure				
High vs. low attractiveness	5.38	0.64	8.36	**<.001**
Negative vs. neutral information	−2.15	0.33	−6.61	**<.001**
Positive vs. neutral information	0.78	0.31	2.48	**.013**
Negative vs. neutral info × high vs. low att	0.56	0.45	1.25	.21
Positive vs. neutral info × high vs. low att	0.84	0.42	2.01	**.04**
*Formula of converging model*	Rating ∼ phase/ information × attractiveness + (1 + phase/ information × attractiveness| subject) + (1 | face)			

*Note. *× stands for interaction. The / in the model formula denotes nesting. *P*-values < .05 are printed in bold.

After information exposure, social-emotional information also affected how attractive participants found the faces (Fig. 5, Table 7). Apart from the main effect of attractiveness, faces associated with positive information were rated as more attractive, and faces associated with negative information were rated as less attractive compared to neutral information, respectively. An interaction of the effect of positive information and attractiveness suggests that the effect of information was more pronounced for the congruent, high attractive faces.

## Discussion

How do facial and moral beauty jointly shape how we judge others? We investigated effects of facial attractiveness and social-emotional person-related information and their interplay on ERPs and social judgments. These global social evaluations should reflect a natural tendency of forming social impressions from visual appearance or semantic information (Todorov et al. 2007, Bliss-Moreau et al. 2008, Goodwin 2015, Uhlmann et al. 2015, Baum and Abdel Rahman 2021). Results show a dominance of moral beauty in valenced social judgments as well as ERPs related to reflexive and evaluative emotional responses (EPN, LPP), whereas facial attractiveness mattered little. In contrast, visual processing (N170) and impressions of attractiveness and likability, that can be formed based on visual information without knowledge about the person, were influenced by both facial attractiveness and social-emotional information.

We found general halo effects of facial and moral beauty in relatively shallow judgments of spontaneous likability and attractiveness. They can be considered shallow or superficial because they can be based on visual impression with no need for additional person information and faces are supposedly processed more as percepts rather than concepts or persons (Schnuerch et al. 2015, Schwartz and Yovel 2016). Consistent with the literature on halo effects, we show that high vs. low attractiveness determined likeability impressions when no other information was available (e.g. Dion et al. 1972, Eagly et al. 1991, Langlois et al. 2000, Zebrowitz and Montepare 2008), and that social-emotional information changed the likability and the attractiveness of faces (Nisbett and Wilson 1977, He et al. 2024). The pattern of results suggests that facial and moral beauty can affect judgments that are not directly related to facial or moral beauty. However, such halo effects may be most pronounced for those relatively shallow judgments.

Importantly, the ratings of attractiveness and likability before and after exposure to social-emotional information show that both factors were successfully manipulated, confirming that faces were clearly perceived as either high or low in attractiveness and that person-related information was perceived either positive, negative, or neutral.

In contrast to the relatively shallow judgments of attractiveness and likeability, we seem less likely to generalize vision-based impressions for explicit social judgments, and more likely to base these judgments on person-related information, rather than facial beauty. Social judgments employed here can be considered less shallow because we asked participants to form person judgments taking all available information into account—that is judgments for which moral character should be most relevant (Goodwin 2015). Results show that only the latencies of social judgments, but not the actual outcomes of the social judgments, were affected by facial attractiveness. Participants hesitated with their judgments when the facial attractiveness was incongruent with the social-emotional information. This suggests that facial beauty was processed during social judgments, even though interestingly, the valence of the judgments was influenced only be the social-emotional information and not by attractiveness.

We investigated the effects on visual face perception in the P1 and N170. The P1 has been associated with generators in the ventral extrastriate cortex (V4) of the fusiform gyrus (Di Russo et al. 2002), while the N170 with the fusiform face area and superior temporal sulcus (Herrmann et al. 2005, Deffke et al. 2007, Nguyen and Cunnington 2014, Gao et al. 2019, Schindler et al. 2023). While the P1 was not modulated, we found enhanced N170 amplitudes for faces of low compared to high attractiveness. This suggests an early and automatic processing of facial attractiveness, presumably related to a modulation of activity in face-selective brain regions. If anything, trends furthermore suggested that this effect was pronounced for positive and negative, but not neutral, associated information. Effects in the N170 suggest enhanced visual processing of faces scoring low in facial beauty. This replicates earlier reports of attractiveness modulations in the N170 (Hahn et al. 2016, Trujillo et al. 2014; but see Revers et al. 2023 for mixed findings for the direction of the effect). It has been suggested that enhanced N170 amplitudes for lower attractiveness could be related to less fluent configural processing compared to higher or average attractiveness (Trujillo et al. 2014). However, in contrast to social-emotional information where we fully counterbalanced the assignment to faces, the attractiveness manipulation entailed purely physical differences in the faces that may have contributed to the N170 modulation, even though luminance was adjusted.

We investigated effects on reflexive emotion processing in the EPN. The EPN has been related to increased extrastriate cortex activation by emotional stimuli content, possibly due to feedback from the amygdala to sensory pathways (Pourtois et al. 2013, Schettino et al. 2016). Our findings show EPN effects of positive and negative social-emotional information independent of attractiveness, suggesting that moral beauty is most relevant to fast and reflexive emotional processing. Although attractiveness as vision-based information could be directly accessed, it did not affect reflexive emotional brain responses during social judgments (Werheid et al. 2007, Schacht et al. 2008). Instead, the EPN results indicate that the nonvisual, social-emotional information mattered most during EPN-generating sensory processes starting at about 200 ms, a time of the processing when visual perception is not yet fully completed and that is largely independent of the current goal or motivation (Schacht and Sommer 2009, Rellecke et al. 2011, 2012).

Finally, we investigated effects on more elaborate and post-perceptual evaluations in the LPP. Broader brain networks underlie the generation of such later ERPs, including widespread cortical, corticolimbic, and subcortical activations associated with visual and emotional processing (Schupp et al. 2007, Liu et al. 2012, Sabatinelli et al. 2013, Trautmann-Lengsfeld et al. 2013). LPP results showed dominant effects of social-emotional information during social judgments, but also suggest that facial attractiveness was processed. Specifically, for faces scoring low in attractiveness, the LPP effect of negative information was slightly more pronounced compared to positive information. This may indicate a compounded effect of negative social-emotional information and low attractiveness during this sustained evaluation of the person. Evidence for similar compounded effects in the LPP was reported for peer opinions of facial beauty and vision-based attractiveness during facial attractiveness judgments (Thiruchselvam et al. 2016). Furthermore, more pronounced LPP amplitudes were related to faster judgments (see SI-page 6 in supplementary material for an additional model with LPP amplitudes as predictor for latencies of social judgments). Consistent with the effects found for latencies and LPP, this relation may indicate that the fast social judgments for congruent attractiveness and social-emotional information were processed more elaborately and efficiently on a neurocognitive level (Schupp et al. 2000).

One may ask whether results were different when the subjective attractiveness rating of each subject for each face was considered. Additional analyses replacing the predefined attractiveness factor with the subjective attractiveness ratings of the participants show, if anything, that social judgments were modulated by subjective attractiveness impressions when the information was positive or neutral (see SI-page 7ff in supplementary material). However, social judgments remained unaffected by either attractiveness scores—consensus or subjective—when the information was negative. Thus, if attractiveness affected social judgments, it was only as a subjective impression and only when person-related information was positive or neutral. This is in line with related findings showing that effects of positive, but not effects of negative emotional information may be modulated by facial appearance or other potentially modulating factors (Baum and Abdel Rahman 2021, Eiserbeck et al. 2023). For ERPs, analyses including subjective attractiveness remained to show the main pattern of results with dominant effects of social-emotional information (SI-page 9ff in supplementary material).

Overall, the current findings show largely similar effects of negative and positive social-emotional information. Nevertheless, the effect of negative may be stronger than the effect of positive social-emotional information possibly related to their intensity and differences in underlying processes which should be further investigated in future studies (please note that pre-ratings of our materials showed that positive and negative information was equally arousing, SI-page 2 in supplementary material). For instance, the effects of positive information may be more susceptible to modulating factors (Isen et al. 1992, Ashby et al. 1999, Baum and Abdel Rahman 2021, Eiserbeck et al. 2023). Furthermore, effects of negative emotions may be less modulated due to a prioritization of potential threat (Öhman and Mineka 2001).

One possible mechanism that can help explain the dominance of moral beauty is emotional arousal. As discussed before, modulations of the EPN and LPP are primarily driven by the arousal dimension of emotions (e.g. Schupp et al. 2003). High and low attractive faces may be similarly arousing (Liu and Chen 2012). In a *post hoc* rating of the faces used in our study, we also show that the high and low attractiveness conditions differ in valence but not in arousal (see SI-page 2 in supplementary material). Moreover, the arousal does not differ between the high/low faces and the medium attractive filler faces, with all conditions showing relatively low arousal ratings on average (M_all_ = 2.48, SD_all_ = 0.97 on a 7-point scale, SI-page 2 in supplementary material). Therefore, emotional responses may prioritize the more arousing social-emotional information as seen in modulations of the EPN and LPP. This dominance is in line with findings employing a similar paradigm with social-emotional information and the same social judgment task (Baum and Abdel Rahman 2020, 2021, Baum et al. 2020; Baum et al. 2024). Thus, our behavioral and neurocognitive findings imply that social judgments and underlying processes involved in social-emotional face and person evaluation rely on the most emotionally arousing information, which is moral beauty, rather than facial beauty.

We cannot rule out the possibility that smaller effects of attractiveness would be reflected in social judgments behaviorally if the scale was more fine-grained. Yet, we also find dominant effects of social-emotional information in ERPs that are more implicit, especially the EPN, which should be minimally influenced by the specific answer scale (e.g. Rellecke et al. 2011). As discussed above, the effects on social judgments seem to be strongly driven by arousal which can help explain the lack of effects of attractiveness on judgments. Importantly, our finding that social-emotional information shows dominant effects is consistent with the literature where appearance-based effects were reduced or absent in face of social-emotional or moral information independent of how fine-grained the scale was (see Mo et al. 2016, Eiserbeck and Abdel Rahman 2020, Eiserbeck et al. 2024).

### Limitations and future directions

Despite carful counterbalancing of the assignment of faces to information, we found unexpected interaction effects of attractiveness and social-emotional information in the ratings before any information was given. We show that this was driven by the neutral information condition and, critically, that there were no effects related to positive or negative information before participants were even exposed to it. Thus, these unexpected modulations seem unproblematic to our key manipulations of positive and negative social-emotional information since these were unaffected.

Our sample consisted of female participants only to avoid confounds due to the different perceptions of attractiveness related to the gender of the participants [see also Tsukiura and Cabeza (2011a, 2011b)]. For this study, we used faces for which the consensus impressions were similar in pre-ratings and the experiment. However, subjective differences in the perception of attractiveness are evident which may depend on e.g. gender and cultures and should be investigated in future studies [see Todorov et al. (2015), Rafiee and Schacht (2023)]. Furthermore, other impressions from facial appearance and their interplay with moral beauty during social judgments should be studied, for example facial trustworthiness (Lischke et al. 2018, Wendt et al. 2019, Weymar et al. 2019, Eiserbeck and Abdel Rahman 2020, Oh et al. 2023).

## Conclusion

Taken together, this study shows that moral beauty, although not physically visible, matters more to our emotional responses and social evaluations of others than visually derived facial beauty. While we do appreciate and process the facial beauty of others and rely on it for shallow judgments, for deeper social judgments we count more on people’s moral beauty than on their facial beauty.

## Supplementary Material

nsae071_Supp

## Data Availability

Data and code are available online (https://osf.io/e529z/).
