# Supplementary material for: A beautiful face is good when we’re judged by others, a moral character is better
Source: Soc Cogn Affect Neurosci. 2024 Oct 17;20(1):nsae071. doi: 10.1093/scan/nsae071 (PMC12036660; doi:10.1093/scan/nsae071)
Supplement: nsae071_Supp [file nsae071_supp.zip › nsae071_Supp.pdf]

Supplementary Information for

A beautiful face is good when we're judged by others, a moral character is better

Julia Baum & Rasha Abdel Rahman

Correspondence: julia.baum@hu-berlin.de

Sample Size

A multiple of three participants were required to counterbalance the assignment of positive, negative, and neutral social-emotional information to faces high or low in attractiveness in our within-subject design. With a planned sample of 24 participants the expected power to find main effects of negative and positive information, of attractiveness, and respective interaction effects as fixed effect regression coefficients in linear mixed model analyses was calculated by simulations (1000 each) using the simr package in R (Green & Macleod, 2016). Accordingly, expected ERP coefficient effects between 0.3 and 0.6 microvolts can be found with power estimates of 85% or higher.

Materials

The selection of high, low, and medium faces was based on pre-ratings of a total of 125 faces (62 male, 63 female) on a 7-point scale (adopted from the Self-Assessment Manikin; Bradley & Lang, 1994, from very attractive (1) to very unattractive (7) by N=15 participants (all female, mean age = 24.67, *SD* = 5.76). As target faces, we selected 12 high attractive faces (6 male, 6 female) that were rated as highest in attractiveness with a low standard deviation, and 12 low attractive faces (6 male, 6 female) that were rated as lowest in attractiveness and a low standard deviation. Additionally, 12 faces of medium attractiveness were selected as fillers (faces with ratings closest to the overall average rating). The high,

low, and medium faces differed in attractiveness ($F(1,28) = 334, p < .001$; $M_{high} = 2.91$, $SD_{high} = .77$, $M_{medium} = 4.21$, $SD_{medium} = .49$, $M_{low} = 6.21$, $SD_{low} = .79$).

Post-hoc, the 36 face stimuli were rated on valence and arousal by an independent sample (N=15, all female, mean age = 26.07, *SD* = 4.20) on 7-point scales (adopted from the Self-Assessment Manikin; Bradley & Lang, 1994). The high, medium, and low attractive faces did not differ in arousal ($F(2,28) = 1.16, p = .33$; M_{all}=2.48, SD_{all}=.97), but they differed in valence ($F(2,28) = 21.61, p < .001$; $M_{high} = 4.39$, $SD_{high} = .62$, $M_{average} = 4.23$, $SD_{average} = .57$, $M_{low} = 3.73$, $SD_{low} = .66$). Low attractive faces were rated more negatively than high attractive faces (b = 0.66, $t(1,14) = 5.53, p < 0.001$), and also than medium attractive faces (b = -0.49, $t(1,14) = -5.56, p < 0.001$). High and medium attractive faces were not rated significantly different (b = 0.16, $t(1,14) = 1.58, p = 0.14$).

A total of 89 short sentences were pre-rated on valence and arousal on 7-point scales from very negative (1) to very positive (7) and not at all arousing (1) to very arousing (7), respectively (adopted from the Self-Assessment Manikin; Bradley & Lang, 1994, by N=15 participants (10 female, 4 male, 1 no gender, mean age = 24.87, *SD* = 5.99). As target information we selected 8 positive, 8 negative and 20 neutral sentences (12 of the neutral sentences were assigned to the filler faces). The pre-ratings confirmed the valence of the information ($F(2,28) = 178, p < .001$; $M_{pos} = 6.23$, $SD_{pos} = .82$, $M_{neg} = 1.71$, $SD_{neg} = .80$, $M_{neu} = 4.17$, $SD_{neu} = .48$). Moreover, pre-ratings showed that positive and negative information was equally arousing ($F(2,28) = 51, p < .001$; positive vs. negative: $t(14) = 1.22$, $p = 0.26$; $M_{pos} = 4.44$, $SD_{pos} = 1.23$, $M_{neg} = 4.86$, $SD_{neg} = 1.10$, $M_{neu} = 1.51$, $SD_{neu} = .71$).

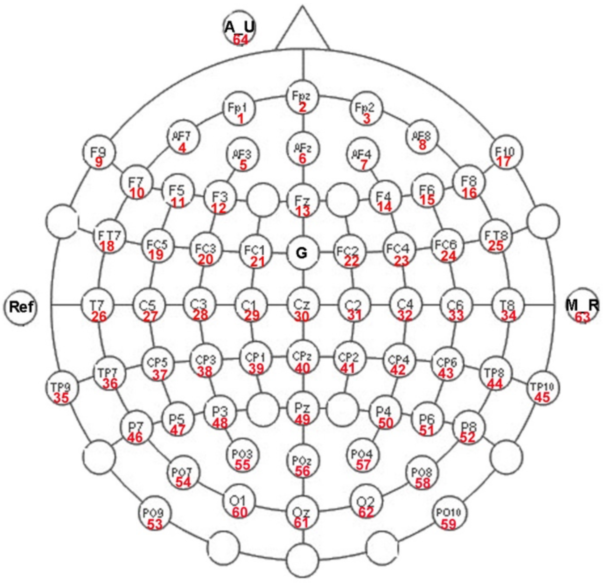

Figure S1: Coverage of sensor positions of our 64 channel montage. The average reference included the 62 scalp channels, meaning it did not include the eye electrode and the right mastoid electrode.

Table S1

Linear mixed model summary statistics and separate comparisons show effects of positive information and negative information each in interaction with attractiveness on the P1.

	**P1**			
Coefficient	*b*	*SE*	*t*	*p*
Intercept (Grand Mean)	2.98	0.61	4.91	**<0.001**
High vs. Low Attractiveness	-0.02	0.12	-0.17	0.86
Negative vs. Neutral Information	-1.28	0.14	-0.09	0.93
Positive vs. Neutral Information	-0.00	0.12	-0.02	0.98
Negative vs. Neutral Info × High vs. Low Att	-0.84	0.25	-0.34	0.73
Positive vs. Neutral Info × High vs. Low Att	-0.30	0.26	-1.14	0.26
Formula of converging model	P1 ~ Information × Attractiveness + (Neg-Neu + Pos-Neu-High-Low || subject) + (1 | face)			

Note. × stands for interaction. Double bars in random effects terms set correlation parameters to zero. Separate comparisons' p-values were fdr corrected for nine tests.

Manipulation checks – Additional analysis before information exposure

Likability rating

An additional model testing the critical information conditions only (positive vs. negative, excluding the neutral condition), showed no effect of positive or negative information before exposure to the information (main effect information (positive vs. negative): b=0.17, *SE*=.23, *t*= .75, *p*=.45, main effect attractiveness: b=2.97, *SE*=0.41, *t*=7.20, *p*<0.001, interaction of information and attractiveness: b=0.18, *SE*=0.43, *t*=0.41, *p*=.68; see SI-Table S2). The neutral condition drove spurious effects of information before exposure, possibly showing that those faces that were randomly assigned to the neutral condition were rated differently by some subjects. Please further note that within the neutral condition, likability ratings were only affected by attractiveness (b=2.71, *SE*=.56, *t*=4.83, *p*<0.001) and were not affected by exposure to neutral information (main effect phase: b=-0.38, *SE*=.20, *t*=-1.93, *p*=.053; interaction of phase and attractiveness: b=-0.05, *SE*=.40, *t*=-0.13, *p*=.90).

Attractiveness rating

An additional model testing the critical conditions only (positive vs. negative, excluding neutral) showed no influence of positive or negative information before exposure information (main effect information (positive vs. negative): b=0.13, *SE*=.21, *t*=0.64, *p*=.52, main effect attractiveness: b=6.43, *SE*=0.60, *t*=10.67, *p*<0.001, interaction of information and attractiveness: b=0.10, *SE*=0.44, *t*=0.24, *p*=.81; see SI-Table S3). Within the neutral condition, attractiveness ratings were only affected by attractiveness (b=5.47, *SE*=.72, *t*=7.59, *p*<0.001) and were not affected by exposure to neutral information (main effect phase: b=-0.34, *SE*=.19, *t*=-1.75, *p*=.08; interaction of phase and attractiveness: b=0.45, *SE*=.39, *t*=1.15, *p*=.25).

Table S2

Mixed model summary statistics show effects of positive vs. negative information (excluding the neutral information condition) each in interaction with attractiveness on ***likability ratings*** *before and after information exposure.*

Positive vs. Negative Information Model				
Coefficient	*b*	*SE*	*z*	*p*
Phase (Before vs. After)	0.56	0.23	2.43	**0.015**
**Before Information Exposure**				
Information (Positive vs. Negative)	0.17	0.23	0.75	0.45
Attractiveness (High vs. Low)	2.97	0.41	7.20	**<0.001**
Attractiveness × Information	0.18	0.43	0.41	0.68
**After Information Exposure**				
Information (Positive vs. Negative)	7.15	0.87	8.21	**<0.001**
Attractiveness (High vs. Low)	2.11	0.48	4.38	**<0.001**
Attractiveness × Information	0.83	0.57	1.45	0.15
Formula of converging model	Rating ~ Phase/Information × Attractiveness + (1 + Phase/ Information × Attractiveness | subject) + (1 | face)			

Table S3

Mixed model summary statistics show effects of positive vs. negative information each in interaction with attractiveness on ***attractiveness ratings*** *before and after information exposure.*

Positive vs. Negative Information Model				
Coefficient	*b*	*SE*	*z*	*p*
Phase (Before vs. After)	0.19	0.15	1.28	0.20
**Before Knowledge Aquisition**				
Information (Positive vs. Negative)	0.13	0.21	0.64	0.52
Attractiveness (High vs. Low)	6.43	0.60	10.67	**<0.001**
Attractiveness × Information	0.10	0.44	0.24	0.81

After Knowledge Aquisition				
Information<br>(Emotional vs. Neutral)	2.86	0.53	5.39	**<0.001**
Attractiveness<br>(High vs. Low)	5.46	0.66	8.29	**<0.001**
Attractiveness × Information	0.30	0.49	0.60	0.55
Formula of converging model	Rating ~ Phase/Information × Attractiveness + (1 + Phase/Information × Attractiveness | subject) + (1 | face)			

Additional Analyses (Discussion)

Table S4

Linear mixed model summary statistics show effects of LPP amplitudes (as centered continuous variable) on the latencies of social judgments.

**Latencies of social judgments (reciprocal transformation -1000/RT)**				
Coefficient	*b*	*SE*	*t*	*p*
Intercept (Grand Mean)	-1.45	0.05	-28.31	**<0.001**
LPP amplitude	-0.01	0.00	-5.79	**<0.001**
Formula of converging model	Latencies ~ LPP<br>+ (LPP || subject) + (1 | face)			

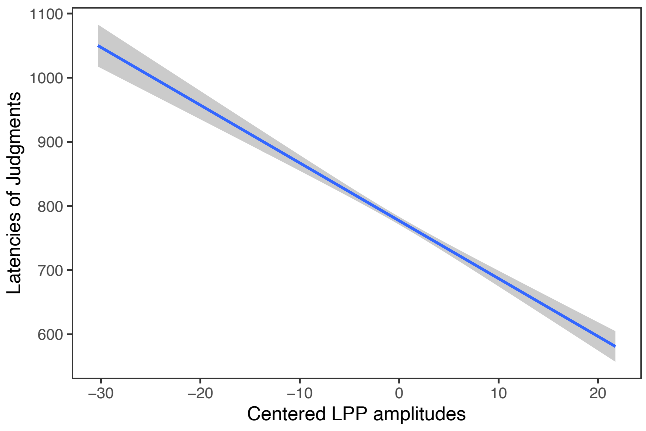

Figure to Table S4. Grey shade shows standard error band.

Individual ratings of attractiveness by each subject for each face as alternative model predictor

Table S5

Predictive accuracy of models in percentage (with standard error) by method of correlation between observed and predicted variables (performance package in R).

**Dependent Variable**	**Model accuracy of original model with predefined factors for attractiveness and information**	**Model accuracy of additional model with the individual rating for attractiveness and the predefined factor for information**
Social judgment	Not applicable for clmm models	
Latencies of social judgment	61.12% (2.00%-points)	61.11% (0.67%-points)
P1	48.56 % (1.06%-points)	48.70% (0.69%-points)
N170	53.14% (3.37%-points)	53.22% (1.06%-points)
EPN	51.12% (1.53%-points)	51.19% (2.19%-points)
LPP	51.44% (1.30%-points)	51.51% (2.07%-points)

Table S6

Mixed model summary statistics and separate comparisons show effects of positive information and negative information each in interaction with ***the individual rating of attractiveness as a covariate*** *(as centered continuous variable), leaving out the predefined factor attractiveness, on social judgments.*

**Social judgments**				
Coefficient	*b*	*SE*	*z*	*p*
Attractiveness Individual Rating (Att.Rating)	0.30	0.05	5.58	**<0.001**
Negative vs. Neutral Information	-8.13	0.47	-17.40	**<0.001**
Positive vs. Neutral Information	6.70	0.48	14.09	**<0.001**
Negative vs. Neutral Info × Att.Rating	-0.48	0.11	-4.14	**<0.001**
Positive vs. Neutral Info × Att.Rating	0.14	0.11	1.26	0.21
Separate comparisons	*b*	*SE*	*z*	*p*

Negative Info: Att.Rating	-0.07	0.11	-0.64	0.52
Positive Info: Att.Rating	0.55	0.10	5.29	**<0.001**
Neutral Info: Att.Rating	0.41	0.05	8.20	**<0.001**
Formula of converging model	Judgments ~ Information × Individual Attractiveness Rating + (Information × Attractiveness | subject) + (1 | face)			

Note. Separate comparisons' p-values were fdr corrected for three tests.

Table S7

Linear mixed model summary statistics and separate comparisons show effects of positive information and negative information each in interaction with ***the individual rating of attractiveness as a covariate*** *(as centered continuous variable), leaving out the predefined factor attractiveness, on latencies of social judgments.*

**Latencies of social judgments**				
Coefficient	*b*	*SE*	*t*	*p*
Intercept (Grand Mean)	-1.44	0.05	-28.39	**<0.001**
Attractiveness Individual Rating (Att.Rating)	-0.005	0.00	-1.39	0.16
Negative vs. Neutral Information	0.008	0.03	0.28	0.78
Positive vs. Neutral Information	0.055	0.03	1.76	0.091
Negative vs. Neutral Info × Att.Rating	0.013	0.00	2.30	**0.024**
Positive vs. Neutral Info × Att.Rating	-0.03	0.00	-6.11	**<0.001**
Separate comparisons	*b*	*SE*	*t*	*p*
Negative Info: Att.Rating	0.01	0.00	2.99	**0.005**
Positive Info: Att.Rating	-0.03	0.00	-6.62	**<0.001**
Neutral Info: Att.Rating	0.001	0.00	0.30	0.76
Formula of converging model	Latencies ~ Information × Individual Attractiveness Rating + (Information × Attractiveness || subject) + (1 | face)			

Table S8

*Linear mixed model summary statistics and separate comparisons show effects of positive information and negative information each in interaction with **the individual rating of attractiveness as a covariate** (as centered continuous variable), leaving out the predefined factor attractiveness, on the P1 amplitudes.*

	**P1**			
Coefficient	*b*	*SE*	*t*	*p*
Intercept (Grand Mean)	2.98	0.61	4.91	**<0.001**
Attractiveness Individual Rating (Att.Rating)	0.01	0.03	0.34	0.74
Negative vs. Neutral Information	-0.009	0.14	-0.07	0.95
Positive vs. Neutral Information	-0.001	0.12	-0.01	0.99
Negative vs. Neutral Info × Att.Rating	-0.004	0.07	-0.07	0.95
Positive vs. Neutral Info × Att.Rating	-0.11	0.07	0.07	0.11
Formula of converging model	P1 ~ Information × Individual Attractiveness Rating + (Neg-Neu + Pos-Neu-High-Low || subject) + (1 | face)			

Table S9

*Linear mixed model summary statistics and separate comparisons show effects of positive information and negative information each in interaction with **the individual rating of attractiveness as a covariate** (as centered continuous variable), leaving out the predefined factor attractiveness, on the N170 amplitudes.*

	**N170**			
Coefficient	*b*	*SE*	*t*	*p*
Intercept (Grand Mean)	-1.49	0.61	-2.45	**0.022**
Attractiveness Individual Rating (Att.Rating)	0.05	0.03	1.25	0.21
Negative vs. Neutral Information	-0.10	0.11	-0.95	0.34
Positive vs. Neutral Information	-0.08	0.13	-0.59	0.56
Negative vs. Neutral Info × Att.Rating	0.10	0.02	1.71	0.091
Positive vs. Neutral Info × Att.Rating	0.08	0.06	1.43	0.153
Formula of converging model		N170 ~ Information × Individual Attractiveness Rating + (Pos-Neu + High-Low + Neg-Neu-High-Low || subject) + (1 | face)		

Table S10

Linear mixed model summary statistics and separate comparisons show effects of positive information and negative information each in interaction with ***the individual rating of attractiveness as a covariate*** *(as centered continuous variable), leaving out the predefined factor attractiveness, on the EPN amplitudes.*

	**EPN**			
Coefficient	*b*	*SE*	*t*	*p*
Intercept (Grand Mean)	-0.11	0.55	-0.20	0.84
Attractiveness Individual Rating (Att.Rating)	-0.08	0.04	-1.98	**0.048**
Negative vs. Neutral Information	-0.88	0.12	-7.50	**<0.001**
Positive vs. Neutral Information	-0.64	0.15	-4.38	**<0.001**
Negative vs. Neutral Info × Att.Rating	0.12	0.07	1.75	0.08
Positive vs. Neutral Info × Att.Rating	0.06	0.06	1.05	0.30
Formula of converging model	EPN ~ Information × Individual Attractiveness Rating + (Information × Attractiveness || subject) + (1 | face)			

Table S11

Linear mixed model summary statistics and separate comparisons show effects of positive information and negative information each in interaction with ***the individual rating of attractiveness as a covariate*** *(as centered continuous variable), leaving out the predefined factor attractiveness, on the LPP amplitudes.*

	**LPP**			
Coefficient	*b*	*SE*	*t*	*p*
Intercept (Grand Mean)	5.96	0.58	10.24	**<0.001**
Attractiveness Individual Rating (Att.Rating)	0.00	0.04	0.20	0.85
Negative vs. Neutral Information	2.04	0.20	10.04	**<0.001**
Positive vs. Neutral Information	1.74	0.16	10.78	**<0.001**
Negative vs. Neutral Info × Att.Rating	-0.16	0.07	-2.36	**0.021**
Positive vs. Neutral Info × Att.Rating	-0.03	0.07	-0.41	0.68
Formula of converging model	LPP ~ Information × Individual Attractiveness Rating + (Information × Attractiveness || subject) + (1 | face)			

Table S12

Mixed model summary statistics show effects of three levels of attractiveness (high, medium, low) for the neutral information condition with separate models for each behavioural measure. Medium attractive faces were filler stimuli that were only paired with neutral information. Since only faces with neutral information are included, the Manipulation Checks (Likability Rating and Attractiveness Rating) contain combined data of pre- and post-information exposure.

**Social Judgment**				
Coefficient	*b*	*SE*	*z*	*p*
High vs. Medium Attractiveness	0.71	0.73	0.97	0.33
Low vs. Medium Attractiveness	-0.62	0.63	-1.00	0.32
Formula of converging model	Judgment ~ Attractiveness + (1 + Attractiveness | subject) + (1 | face)			

**Latencies of Social Judgment**				
Coefficient	*b*	*SE*	*t*	*p*
Intercept (Grand Mean)	-1.47	0.06	-22.86	**<0.001**
High vs. Medium Attractiveness	0.03	0.03	1.06	0.30
Low vs. Medium Attractiveness	-0.00	0.03	-0.05	0.92
Formula of converging model	Judgment ~ Attractiveness + (1 + Attractiveness | subject) + (1 | face)			

**Likability Rating (Manipulation check)**				
Coefficient	*b*	*SE*	*z*	*p*
High vs. Medium Attractiveness	1.11	0.45	2.44	**0.015**
Low vs. Medium Attractiveness	-1.28	0.44	-3.11	**0.002**
Formula of converging model	Rating ~ Attractiveness + (1 + Attractiveness | subject) + (1 | face)			

**Attractiveness Rating (Manipulation check)**				
Coefficient	*b*	*SE*	*z*	*p*
High vs. Medium Attractiveness	2.03	0.45	4.49	**<0.001**
Low vs. Medium Attractiveness	-3.25	0.46	-7.00	**<0.001**
Formula of converging model	Rating ~ Attractiveness + (1 + Attractiveness | subject) + (1 | face)			

Table S13

Linear mixed model summary statistics show effects of three levels of attractiveness (high, medium, low) for the neutral information condition with separate models for each ERP. Medium attractive faces were filler stimuli that were only paired with neutral information.

**P1**				
Coefficient	*b*	*SE*	*t*	*p*
Intercept (Grand Mean)	2.26	0.71	3.20	**0.004**
High vs. Medium Attractiveness	0.03	0.19	0.15	0.88
Low vs. Medium Attractiveness	-0.059	0.20	-0.30	0.76
Formula of converging model	P1 ~ Attractiveness + (1 | subject) + (1 | face)			

**N170**				
Coefficient	*b*	*SE*	*t*	*p*
Intercept (Grand Mean)	-1.42	0.60	-2.36	**0.026**
High vs. Medium Attractiveness	0.064	0.23	0.28	0.78
Low vs. Medium Attractiveness	-0.16	0.23	-0.69	0.50
Formula of converging model	N170 ~ Attractiveness + (1 | subject) + (1 | face)			

**EPN**				
Coefficient	*b*	*SE*	*t*	*p*
Intercept (Grand Mean)	0.45	0.52	0.89	0.38
High vs. Medium Attractiveness	-0.27	0.37	-0.73	0.47
Low vs. Medium Attractiveness	-0.12	0.37	-0.32	0.75
Formula of converging model	EPN ~ Attractiveness + (1 | subject) + (1 | face)			

**LPP**				
Coefficient	*b*	*SE*	*t*	*p*
Intercept (Grand Mean)	4.66	0.49	9.46	**<0.001**
High vs. Medium Attractiveness	0.23	0.25	0.93	0.36
Low vs. Medium Attractiveness	0.03	0.25	0.11	0.92
Formula of converging model	LPP ~ Attractiveness + (1 | subject) + (1 | face)			

References

Bradley, M. M., & Lang, P. J. (1994). Measuring emotion: The self-assessment manikin and the semantic differential. *Journal of Behavior Therapy and Experimental Psychiatry*, *25*(1), 49–59. https://doi.org/10.1016/0005-7916(94)90063-9

Green, P., & Macleod, C. J. (2016). SIMR: An R package for power analysis of generalized linear mixed models by simulation. *Methods in Ecology and Evolution*, *7*(4), 493–498. https://doi.org/10.1111/2041-210X.12504
